# Biochar aided priming of carbon and nutrient availability in three soil orders of India

**DOI:** 10.1038/s41598-024-56618-w

**Published:** 2024-04-10

**Authors:** T. J. Purakayastha, Tanumoy Bera, Saptaparnee Dey, Pooja Pande, Savita Kumari, Arpan Bhowmik

**Affiliations:** 1 Division of Soil Science and Agricultural Chemistry, ICAR-Indian Agricultural Research Institute, New Delhi, 110012 India; 2 Texas A&M AgriLife Research Center, Beaumnt, TX 77713 USA; 3 Division ICAR-Indian Agricultural Research Institute, Dirpai Chapori, Gogamukh, Dhemaji, Assam 787035 India

**Keywords:** Alfisol, Biochar, Carbon mineralization, Inceptisol, Mollisol, Nutrient availability, Biogeochemistry, Environmental sciences

## Abstract

In recent years biochar (BC) has gained importance for its huge carbon (C) sequestration potential and positive effects on various soil functions. However, there is a paucity of information on the long-term impact of BC on the priming effect and nutrient availability in soil with different properties. This study investigates the effects of BC prepared from rice husk (RBC4, RBC6), sugarcane bagasse (SBC4, SBC6) and mustard stalk (MBC4, MBC6) at 400 and 600 °C on soil C priming and nitrogen (N), phosphorus (P), and potassium (K) availability in an Alfisol, Inceptisol, and Mollisol. BC properties were analyzed, and its decomposition in three soil orders was studied for 290 days in an incubation experiment. Post-incubation, available N, P, and K in soil were estimated. CO_2_ evolution from BC and soil alone was also studied to determine the direction of priming effect on native soil C. Increasing pyrolysis temperature enhanced pH and EC of most of the BC. The pyrolysis temperature did not show clear trend with respect to priming effect and nutrient availability across feedstock and soil type. MBC6 increased C mineralization in all the soil orders while RBC6 in Alfisol and SBC6 in both Inceptisol and Mollisol demonstrated high negative priming, making them potential amendments for preserving native soil C. Most of the BC showed negative priming of native SOC in long run (290 days) but all these BC enhanced the available N, P, and K in soil. SBC4 enhanced N availability in Alfisol and Inceptisol, RBC4 improved N and P availability in Mollisol and P in Alfisol and MBC6 increased K availability in all the soils. Thus, based on management goals, tailored BC or blending different BC can efficiently improve C sequestration and boost soil fertility.

## Introduction

Biochar (BC) gained importance in modern agricultural and environmental management since the identification of the so-called *terra preta* by the late Wim Sombroek in the Amazon, Brazil. The Amazonian *terra preta* soils are more fertile than the surroundings which is believed to be due to the BC additions from “slash and char” agricultural practices^1–3^. Nowadays BC has become a prime research focus owing to its exceptional and distinctive characteristics. Application of BC could be a win-win strategy to sequester carbon (C), augment soil fertility, reduce leaching, reclaim acid soils and degraded lands^4–6^. BC carbon being aromatic in nature can potentially persist over a long period in cultivated and natural ecosystems. Thus, BC is biologically and chemically resistant when applied to soil^4,5^ and has the potential to mitigate global warming^7,8^. Yet BC application can affect native soil organic matter (SOM) decomposition through so-called “priming effects” (PEs), which ultimately leads to changes in soil organic carbon (SOC) mineralization rates^9–11^. When BC is added to soils, it can either serve as a source or a sink of ecosystem C depending on the interactions between BC, microbes, and non-BC SOM^11^. Myriad of studies defined priming effect as the alterations in the mineralization of native SOM triggered by the addition of new substrate^12^. In this regard, it was reported that the effect of BC on priming and C sequestration could be governed by global warming^13^. The direction of priming effect could be positive (accelerated mineralization of SOM)^14,15^, or negative (retarding soil SOM decomposition)^11,16,17^. Positive priming effect may happen due to increased mineralization of resistant SOM components when stimulated by the addition of BC which fuels the microorganisms with mineralizable C, nitrogen (N), phosphorus (P) and micronutrients^11,12,18^. Presumably, on the contrary, due to its porous nature and high affinity for SOM^19,20^, BC has the capability to sequester non-BC SOM within its pore network, providing a physical barrier against enzymatic degradation and abiotic oxidants showing negative priming effect^21^. It was reported that adsorption of SOM, encapsulation of SOM and microbial enzymes, soil aggregate formation, and the decrease in mineralization due to the release of harmful compounds might be the mechanisms of negative priming^18^. A prior study cited a 15% rise in C mineralization after BC application, showcasing a positive priming within a relatively brief timeframe^14^. Nevertheless, another study reported negative priming effects persisting for a six-month period after BC application^16^. Furthermore, in a 120-day incubation experiment, findings revealed that amendments with rice husk, pecan shell, and bamboo BC resulted in an initial negative priming on SOM mineralization after 15 days and it increased with time^22^. However, contradictory findings on priming effects also have been documented in literature^11,23,24^. The BC decomposition rate vis-à-vis direction and magnitude of priming of SOC may vary with pyrolysis temperature^10,16,23^, BC aging, experimental duration^11,12,24,25^, soil clay and C contents^12,16,26,27^, type of feedstock^28,29^ and pyrolysis conditions^16,30^. It has been reported that with an increase in pyrolysis temperature, positive priming of SOC generally decreases^11,14,31^. Furthermore, low temperature BC is easily degraded as compared to high temperature BC^32–34^. This may be because high-temperature BC contains lower amounts of labile C^15,30^. Besides, greater sorption of native SOC to the higher temperature BC with a higher specific surface area and porosity may block microbial extracellular enzymes from accessing SOC, thus lowering positive priming or stabilizing SOC^23^. Overall, BC generally results in short-term positive priming on SOC mineralization, but longer-term C stabilization (i.e., negative priming), possibly linking to (i) the depletion of labile C over time and (ii) the decreased microbial accessibility to SOC due to the physicochemical interaction with BC^31,35^. In this connection, the direction of the primed SOC mineralization exhibited three distinct stages as positive-negative-positive in the BC (300 °C) treated soil, while the opposite direction at each stage occurred in the BC (500 °C) treated soil^10^ Besides this, SOM and clay content are also determinant factors for the positive and negative priming of BC. In this regard, a study clearly demonstrated that the BC prepared from the wood of *Eucalyptus saligna* at 500 °C caused smaller positive priming in the clay-poor Inceptisol or negative priming in the clay-rich Entisol, Oxisol and Vertisol^31^. In contrast, the presence of complex organic matter (OM) such as humic acid may have an inhibitory effect on the mineralization of BC carbon over short and long time periods^36^. This phenomenon is attributed due to substrate switching mechanism. Mollisol with higher clay and SOM contents showed a negative priming effect while Ultisol with low clay and SOM contents showed a positive priming effect^23^. The same BC, therefore, behaved differently due to differences in soil properties. Still confusion exists whether the short- and long-term effects that BC amendment will have on soil C decomposition and sequestration^11,12^. There is also a great deal of discrepancy between studies that find a positive versus negative priming effect may lie in the materials used, including soil, BC type, experimental conditions e.g., water saturation, atmosphere, or time frame.

On the other hand, BC priming effect may influence nutrient cycling and thereby affect the availability of nutrients which are locked up in SOM. In this connection, BC is reported to enhance soil fertility directly by providing essential soil nutrients and soil organic/inorganic carbon^28,37^ or indirectly by neutralizing soil acidity^38,39^. The addition of BC can stimulate microbial activity, retain soil nutrients, immobilize toxic contaminants, and improve soil physicochemical properties such as cation exchange capacity (CEC), water holding capacity (WHC), and soil aeration^40–43^. BC being alkaline in nature is reported to enhance pH of acid soil which results in increased availability of nutrients^44–48^. The maize BC enhanced the available N and P, while wheat BC increased the available potassium (K) content in an Inceptisol^49^. Typically, P appears more available in soils to which BC has been applied^50,51^ and P sorption rates to the surface of ferrihydrite were markedly decreased in the presence of BC^52^. Thus, a more prominent improvement in soil fertility can be achieved by BC application to sandy soil having low clay, nutrient, and SOM contents^28^. Thus, most of the studies concentrated on BC aided priming of native SOC or nutrient availability separately but the main question which is unanswered is impact of SOC priming on available nutrients in soil.

**Figure 1:**
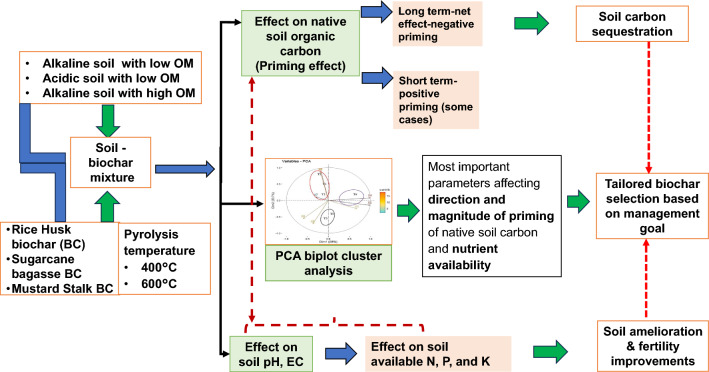
Diagrammatic representation of biochar aided priming of carbon and nutrient availability in three soil orders of India.

It is hypothesized that BC prepared from three selected feedstock and two pyrolysis temperatures having varying properties might influence the direction and magnitude of mineralization and priming of native SOM and nutrient availability in three distinct soil orders namely an Alfisol, Inceptisol, and Mollisol. The first objective of the study was to compare the physical, chemical, and physicochemical properties of BC and linking these to explain the BC aided priming of SOC in three soil orders with varying physico-chemical properties. The second objective was to study the long-term impact of BC on available N, P, and K contents in soil as influence by BC aided priming (Fig. 1).

## Material and methods

### Biochar (BC) production and characterization

Biochar (BC) was prepared from rice husk (R), sugarcane bagasse (S), and mustard stalk (M) in an electrically operated small pyrolysis chamber at 400 °C (4) and 600 °C (6) in a nitrogen environment (1.5 L min^−1^) to obtain six BC with distinct feedstock and characteristics (RBC4, RBC6, MBC4, MBC6, SMC4, and SBC6). The biomass was put inside a closed steel chamber and the temperature was raised at 2 °C min^−1^ until the final temperature reached 400 °C or 600 °C and held constant for 2 h with the help of a digital controller as per the established protocol^48,53^. After 2 h, the pyrolysis chamber was switched off to cool down naturally at ambient condition while maintaining the nitrogen flow. On the next day, the BC was collected and stored in a sealed plastic container. For the incubation experiment, the BC samples were ground in a mixer grinder followed by passing these samples through a 0.1 mm sieve.

BC was characterized for pH and EC^54^, volatile matter and ash contents^55^, calcium carbonate equivalent (CCE)^53^, bulk density (BD)^56^, particle density (PD)^57^, and porosity (1-BD/PD). C, hydrogen (H), and N contents of the BC were measured by dry combustion method in an Elemental Analyzers capable of measuring C, H and N contents^58^ (Eurovector, Pavia, Italy).

### Soil

Three contrasting soil orders i.e., Inceptisol (alkaline soil with low SOM), Alfisol (acidic soil with SOM), and Mollisol (alkaline soil with high SOM) were selected for their distinct organic C contents and physico-chemical properties. Inceptisol, Alfisol, and Mollisol were collected to a depth of 0–15 cm from experimental farms of Indian Agricultural Research Institute, Delhi, Birsa Agricultural University, Ranchi, Jharkhand and Govind Ballabh Pant University of Agriculture and technology, Pantnagar, Uttarakhand, respectively in India. The soil samples were dried in a shade, crushed with a wooden pestle and mortar, passed through a 2-mm sieve and stored at room temperature. For determination of soil C, the soil samples were passed 0.5 mm sieve. The basic physico-chemical properties of soils were measured using standard protocol^58–62^ as mentioned in Table 1.

**Table 1:** Physico-chemical properties of soils.

Properties	Alfisol-Ranchi	Inceptisol-Delhi	Mollisol-Pantnagar	References
Sand	55.0	69.4	32.0	
Silt	22.0	13.7	39.0	
Clay	23.0	16.9	29.0	
Texture	Sandy clay loam	Sandy loam	Silty Clay loam	Bouyoucos (1951)^59^
pH	5.9	8.0	7.4	Jackson (1973)^60^
EC (dS m^−1^)	0.29	0.70	0.40	Jackson (1973)^60^
Soil organic C (g kg^−1^)	5.14	4.11	8.58	Tabatabai and Bremner (1970)^59^
Dissolved organic C (g kg^−1^)	0.31	0.34	0.60	Agnelli et al. (2016)^61^
Maximum water holding capacity (%)	23.9	27.7	28.5	
Soil classification	Typic Haplustalf	Typic Haplustept	Aquic Hapludoll	Soil Survey Staff (2014)^62^

### Incubation experiment

**Figure 2:**
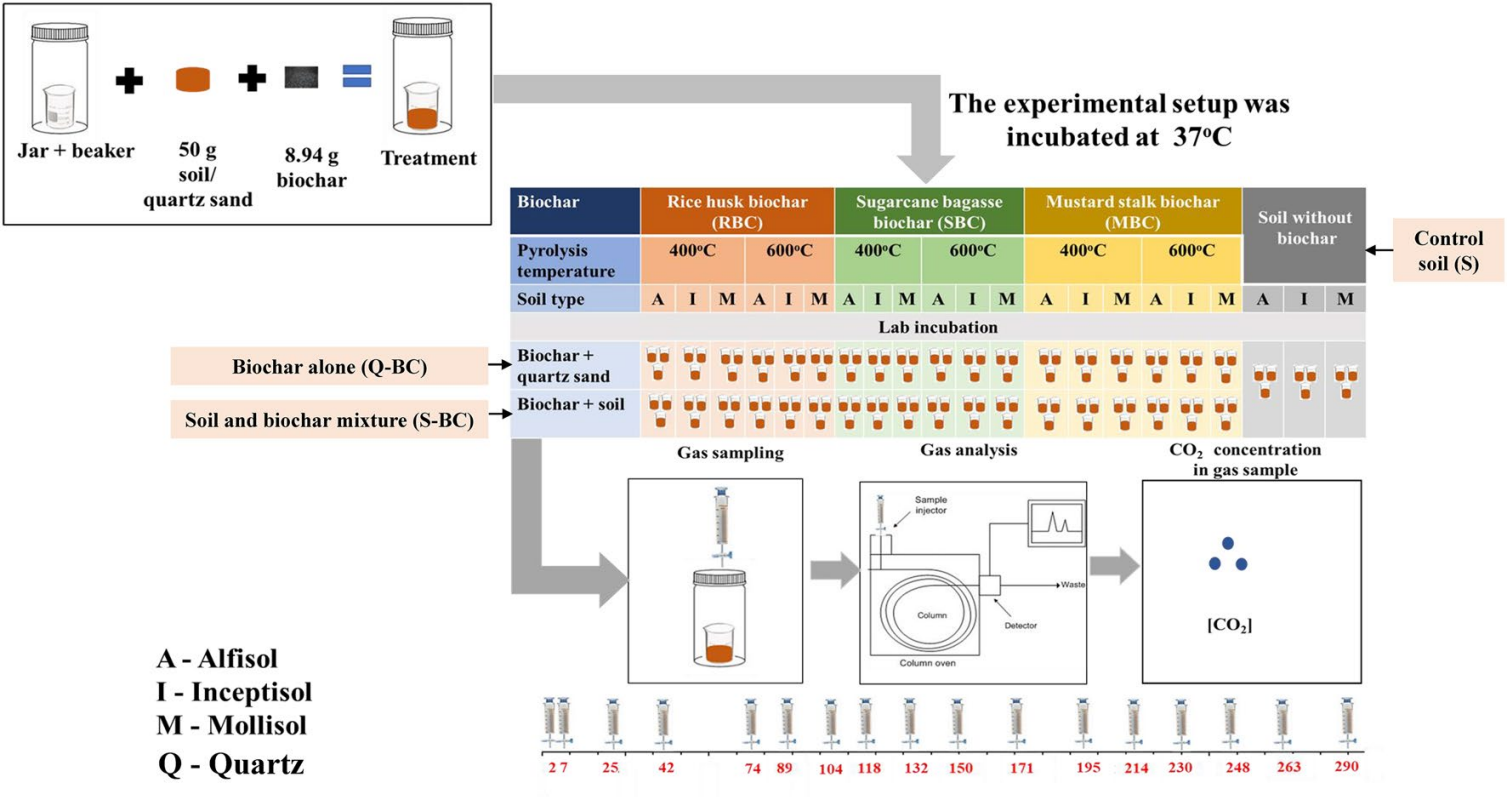
Schematic diagram of the 290-day laboratory experiment for determining priming effect.

A laboratory incubation priming effect experiment was conducted including combinations of three soils, six BC produced from three feedstocks at two temperatures. The experiments included 63 units (Fig. 2). Among them, one set is soil alone treatment i.e. control (S) (soil where no BC was added) for each soil order. In another set three orders of soils were mixed with six BC (S-RBC4, S-RBC6, S-SBC4, S-SBC6, S-MBC4 and S-MBC6), each treatment was replicated thrice (Total = 6 BC * 3 soil orders*3 replication = 54). However, in the soil-BC mixture (S-BC), a portion of the BC undergoes mineralization, resulting in CO_2_ production. Yet, our study specifically aimed to assess the influence of BC addition on the mineralization of native SOM which is termed as priming. To isolate the CO_2_ evolution from BC alone, a separate set of BC and quartz (Q-BC) experiments were conducted, involving six BC types (RBC4, RBC6, SBC4, SBC6, MBC4, and MBC6). CO_2_ readings from Q-BC were subtracted from S-BC to determine the CO_2_ evolution from mineralization of native soil SOM only. Each BC alone treatment (Q-BC) included the addition of 10 ml soil solution (as microbial culture for all soil types) to mimic the environment of soil-BC mixture (S-BC) for each soil type and BC. In Q-BC, each treatment was replicated thrice which resulted in 54 units (6 BC*3 corresponding soil extracts*3 replication) Additionally, 3 jars without soil/quartz (blank) were maintained to subtract CO_2_ concentration in the head space atmosphere of the jar. Thus, the experiment included 120 [54 (S-BC) + 54 (Q-BC) + 9 (S) + 3 (Blank)] incubation units to determine the priming effect. For soil alone and S-BC experiment, 50 g air dry soil was taken in 50 ml capacity beaker. In S-BC experiment, 8.94 g BC was added, thoroughly mixed, required amount of distilled water (16.0 ml for Alfisol, 19.0 ml for Mollisol, and 18.0 ml for Inceptisol) was added to maintain moisture at 67% of maximum water holding capacity. In soil alone treatment i.e. control soil (S) BC was not added. The beakers were placed in 500 ml Mason jars and kept in an incubator at 37 °C. In BC alone experiment i.e. Q-BC treatment, 50 g acid washed quartz sand along with 8.94 g BC was taken in 50 ml beaker, 10 ml soil solution (as microbial culture for all soil type), and required amount of N [(NH_4_)_2_SO_4_], P (KH_2_PO_4_), and K (KH_2_PO_4_ balanced by KCl) (6 ml for Alfisol, 8 ml for Inceptisol and 6 ml for Alfisol) was added, placed in 500 ml Mason jars and kept in the incubator at the same temperature as above. Microbial inoculation was prepared separately from three fresh moist soils by adding 100 ml of distilled water in fresh moist soil (10 g oven dry weight basis), shaken in a mechanical shaker for 5 hours and filtered by Whatman No. 42 filter paper. In each Alfisol incubation unit, N P, and K were applied at 5.36, 0.142, and 3.35 mg, respectively. Similarly, N, P and K rates were 3.19, 0.109, and 2.05 mg, and 6.25, 0.164, and 2.98 mg for Inceptisol and Mollisol, respectively. The nutrient rates were decided based on available N^63^, P^64^, and K^65^ contents in the respective soils. The moisture content of soil or S-BC mixture was maintained at 67% maximum water holding capacity throughout the incubation. The individual beakers (50 mL) either containing control soil (S), BC only (Q-BC) or soil with BC mixture (S-BC) were placed inside airtight Mason jars (500 mL capacity) with lid containing a rubber septum. The jars were incubated in a BOD incubator at 37 °C temperature in dark. On previously determined days during the incubation period, an aliquot of 5 mL of gas sample was drawn from each jar with the help of a syringe (Agilent 4890, USA), and injected to a gas chromatograph (Agilent, model 4890, USA) with packed column (Porapak Q) and TCD detector. On each sampling days, after analysis of the gas samples, jars were opened to ventilate with compressed fresh air generated by a laboratory pump for one minute for aeration. In case of any moisture loss detected by weighing the incubation unit (Initial weight − final weight), same amount of distilled water was added to attain the initial weight for maintaining the moisture content. CO_2_ concentration in gas samples were quantified from standard curve prepared for each sampling dates. The standard curves were prepared by volumetric dilutions (100, 200, 300, 400, 500 µL) of commercially available 1.10% CO_2_ standard gas balance by air obtained from Laser Gases, India, with Norwegian Accreditation No. QUAL 013. The CO_2_-C concentration was expressed in mg per kilogram of soil basis. The amount of CO_2_ evolved was estimated on 2, 7, 25, 42, 74, 89, 104, 118, 132, 150, 171, 195, 214, 230, 248, 263, 290 days of incubation. The cumulative CO_2_-C evolved was calculated by summing the CO_2_-C evolution data in each sampling days using an additive approach. The priming effect (PE) of native SOC induced by BC was calculated as follows^66^:

$$PE=\left[C{\text{min}} S-BC mixture \right]-\sum \left[(C{\text{min}} Q-BC )+(C{\text{min}} S)\right]$$

where *C*min is amount (mg kg^−1^ soil) of cumulative CO_2_-C evolved over 290 days of incubation, S-BC means soil and biochar mixture, Q-BC means biochar alone treatment i.e. quartz and biochar along with soil solution (as microbial culture for respective soil type), S means control soil. The positive value of PE indicates positive priming and the negative value indicates negative priming.

The positive value of PE indicates loss of native SOC through mineralization, while the negative value indicates gain of native SOC through accretion in the presence of BC. After the end of the incubation period, the available N^63^, P^64^, and K^65^ contents in soils were measured.

### Statistical analysis

The data encompassing C mineralization, pH, EC, and available N, P, K contents in control soil (S), BC alone (Q-BC), the arithmetic sum of control soil (S) and BC alone (S + Q-BC), and soil with BC mixture (S-BC) over 290 days underwent one-way ANOVA. Post hoc analysis using Duncan’s multiple range test at a 5% significance level was performed to distinguish the means (conducted in SPSS version 20.0). Additionally, a Pair T-test (*p* = 0.05) was employed to differentiate the mean values of cumulative C mineralization data between S + Q-BC and S-BC mixtures (conducted in SPSS version 20.0). Principal Component Analysis (PCA) using R software)^67^ was carried out to discern the contribution of various factors to the priming effects of soil organic matter and to segregate homogeneous treatments based on BC feedstock and pyrolysis temperature.

## Results

### Chemical and physical characterization of biochar (BC)

The yield of BC prepared from rice husk, sugarcane bagasse and mustard stalk at 400 and 600 °C varied between 45 and 50% and the chemical properties of all BC differed significantly (Table 2). The pH of BC increased by on an average 1.25 times due to increase in pyrolysis temperature and SBC6 (pH 11.4) being at par with MBC6 (pH 11.0) showed the highest pH. The electrical conductivity (EC) of SBC and MBC increased significantly due to increase in pyrolysis temperature, while it did not influence the EC of RBC. Among the BC, MBC showed higher EC than either RBC or SBC. The calcium carbonate equivalent (CCE) increased significantly across all the BC with increase in pyrolysis temperature with the highest CCE recorded in MBC6 (34.4%) followed by SBC6 (24.1%). The C (all BC) and N contents (except SBC) increased significantly, while H, O, and H:C and O:C atom ratio decreased with increase in pyrolysis temperature. The C content was higher in SBC (69.4%), while MBC was richer in N content (1.51%). The C: N decreased in RBC while increased in SBC with increase in pyrolysis temperature. The water-soluble P and K contents in BC in general increased with increase in pyrolysis temperature. The MBC was very rich in K (23.2 mg kg^−1^), while the RBC was rich in P (0.93 mg kg^−1^). In general, the volatile matter decreased, and ash contents increased in BC with increasing pyrolysis temperature. The volatile matter was highest in SBC while the ash content was highest in RBC. The bulk density did not show clear cut trend with respect to pyrolysis temperature while the particle density and porosity significantly increased (except SBC).

**Table 2:** Characterization of biochar.

	RBC		SBC		MBC	
	400 °C	600 °C	400 °C	600 °C	400 °C	600 °C
pH	8.22§^c^	9.72^b^	8.98^bc^	11.4^a^	8.47^c^	11.0^a^
EC (ds m^−1^)	0.22^d^	0.21^d^	0.20^d^	0.58^c^	2.41^b^	4.76^a^
CCE (%)	16.0^d^	18.5^c^	19.7^c^	24.1^b^	19.1^c^	34.4^a^
C (%)	47.4^d^	53.7^c^	61.4^b^	69.4^a^	58.4^b^	66.5^a^
H (%)	3.26^b^	2.19^d^	3.94^a^	2.99^c^	3.91^a^	2.26^d^
N (%)	0.91^d^	1.18^c^	0.83^f^	0.86^ef^	1.37^b^	1.51^a^
O (%)	22.9^a^	6.13^d^	18.8^b^	2.40^e^	21.2^a^	9.96^c^
H:C (atom ratio)	0.82^a^	0.49^b^	0.77^a^	0.52^b^	0.80^a^	0.41^c^
O:C (atom ratio)	0.36^a^	0.09^d^	0.23^c^	0.03^e^	0.27^b^	0.11^d^
C:N	52:1^c^	46:1^d^	74:1^b^	81:1^a^	43:1^d^	44:1^d^
WSK (g kg^−1^)	0.71^d^	1.60^d^	2.14^d^	4.41^c^	15.6^b^	23.2^a^
WSP (g kg^−1^)	0.38^b^	0.93^a^	0.11^e^	0.18^d^	0.18^d^	0.08^c^
Volatile matter (%)	24.6^b^	14.8^d^	30.4^a^	19.5^c^	26.7^b^	10.5^e^
Ash (%)	26.1^b^	36.8^a^	15.1^d^	24.3^b^	15.1^d^	19.7^c^
Bulk density (Mg m^−3^)	0.27^a^	0.27^a^	0.10^d^	0.13^c^	0.14^bc^	0.15^b^
Particle density (Mg m^−3^)	0.51^c^	0.86^b^	0.27^e^	0.38^d^	0.40^d^	0.65^b^
Porosity (%)	47.7^d^	68.5^b^	60.8^c^	65.0^bc^	66.1^b^	76.6^a^

^§^The data followed by different lower-case letters in a particular parameter in each row are significantly different according to Duncan’s Multiple Range Test at *P* = 0.05.

*RBC* rice husk biochar, *SBC* sugarcane bagasse biochar, *MBC* mustard stalk biochar, *EC* electrical conductivity, *CCE* calcium carbonate equivalent, *C* carbon, H hydrogen, *N* nitrogen, *O* oxygen, *WSK* water soluble potassium, *WSP* water soluble phosphorus.

### Decomposition of biochar (BC) in soil

**Figure 3:**
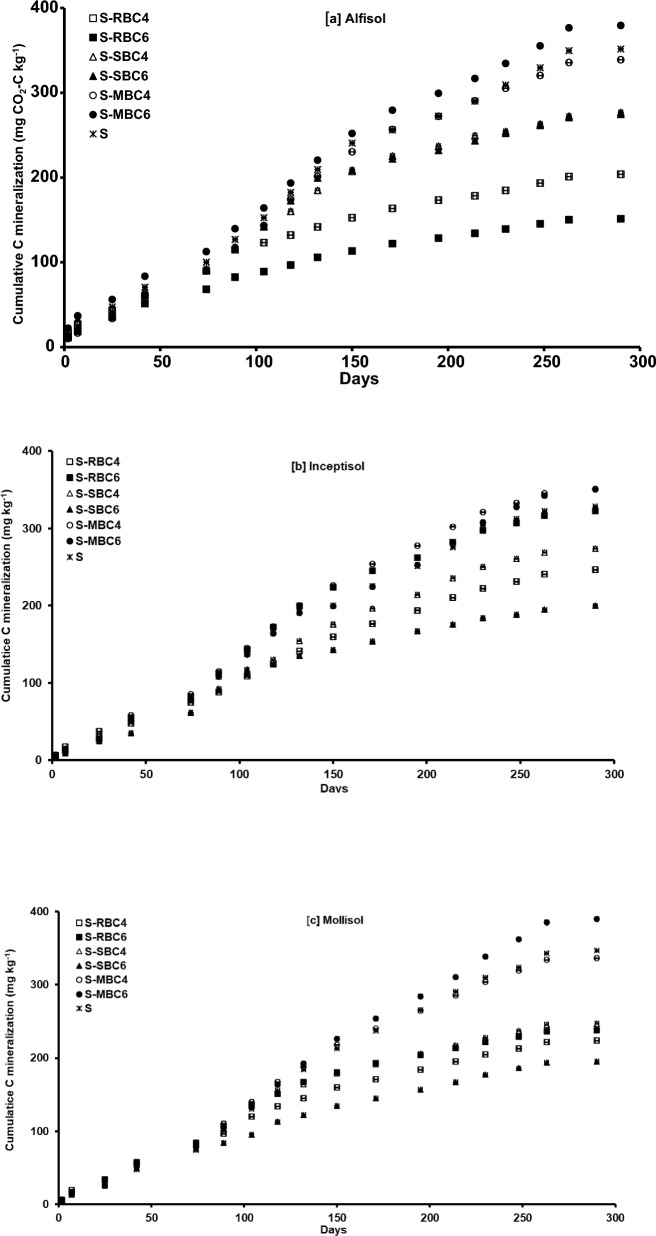
Effect of feedstock and pyrolysis temperature of biochar on cumulative carbon mineralization in (**a**) Alfisol, (**b**) Inceptisol, (**c**) Mollisol during 290 days of incubation. S-Control soil; S-RBC4-Soil with rice husk biochar prepared at 400 °C; S-RBC6-Soil with rice husk biochar prepared at 600 °C; S-SBC4-Soil with sugarcane bagasse biochar prepared at 400 °C; S-SBC6-soil with sugarcane bagasse biochar prepared at 600 °C; S-MBC4-soil with mustard stalk biochar prepared at 400 °C; and S-MBC6-soil with mustard stalk biochar prepared at 600 °C. Each data point represents arithmetic mean of replicates with standard error.

The stability of BC C was assessed by cumulative C loss through mineralization. The cumulative C mineralization (CumC_min_) evolved from all the BC, except MBC in Alfisol, increased rapidly up to 100 to 150 days and thereafter it levelled off for the rest of the period (Fig. 3a). As the time elapsed, the difference between the treatments in terms of CumC_min_ became widened. The CumC_min_ from MBC6 was higher than the rest of the BC and at the end of the incubation experiment CumC_min_ from MBC6 was higher to the tune of 7.88% over control. However, all other BC i.e. MBC4, SBC4, SBC6, RBC4, and RBC6 showed lower CumC_min_ as compared to control. The CumC_min_ was recorded lowest in RBC6, followed by RBC4, SBC4, SBC6 and MBC4.

Throughout the incubation period, the MBC4 and MBC6 being at par with each other showed higher CumC_min_ than the other treatments in Inceptisol (Fig. 2b). As compared to control, the CumC_min_ from all the BC except MBC4 and MBC6 was lower. MBC4 and MBC6 recorded 6.68 and 6.52% higher CumC_min_ over control respectively, at the end of the experiment. The SBC6 showed the lowest CumC_min_ over the incubation period, especially after 132 days and CumC_min_ from SBC6 was 43.0% lower than MBC4 after the incubation experiment. The CumC_min_ from RBC4 was second lowest followed by SBC4, and RBC6.

In Mollisol, the CumC_min_ from all the BC treatments and control were at par up to 74 days of incubation and thereafter the treatments started segregating (Fig. 3c). The MBC4 and control were almost at par with each other in terms of CumC_min_. The CumC_min_ from SBC4 and RBC6 was almost same up to 230 days of incubation and thereafter, the former showed marginally higher CumC_min_ than the latter. The CumC_min_ was invariably lower in SBC6 than the rest of the treatments after 89 days of incubation for the remaining period. The CumC_min_ from RBC4 was second lowest after 114 days of incubation. At the end of the incubation period i.e. at 290th day, all the BC except MBC6 recorded lower CumC_min_ as compared to control while MBC6 showed 12.4% higher CumC_min_ than that of control_._

### Biochar (BC) priming effects

**Figure 4:**
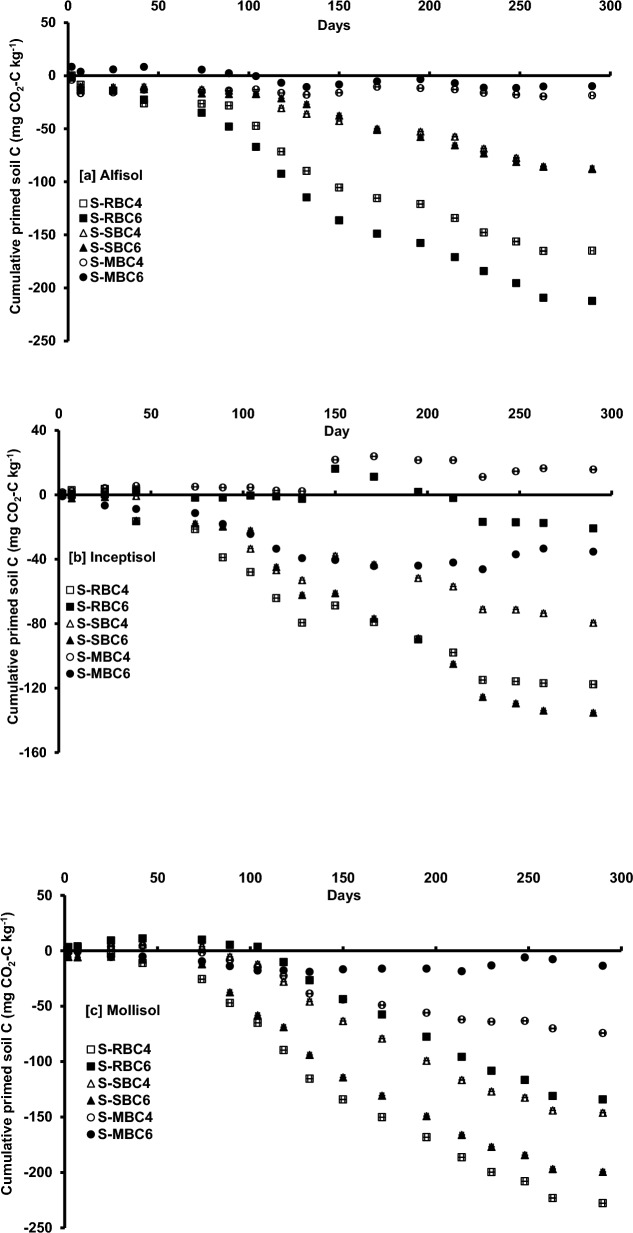
Effect of feedstock and pyrolysis temperature of biochar on cumulative priming of native soil organic carbon in (**a**) Alfisol, (**b**) Inceptisol, and (**c**) Mollisol during 290 days of incubation. S-RBC4-Soil with rice husk biochar prepared at 400 °C; S-RBC6-Soil with rice husk biochar prepared at 600 °C; S-SBC4-Soil with sugarcane bagasse biochar prepared at 400 °C; S-SBC6-soil with sugarcane bagasse biochar prepared at 600 °C; S-MBC4-Soil with mustard stock biochar prepared at 400 °C; and S-MBC6-Soil with mustard stock biochar prepared at 600 °C. Each data point represents arithmetic mean of replicates with standard error.

The trend of native SOC priming by BC in Alfisol, Inceptisol and Mollisol continued throughout the incubation period, albeit the direction and magnitude changed over time (Fig. 4a, b, and c). In Alfisol, initially the positive priming continued in MBC6 up to 89 days of incubation and thereafter negative priming of native SOC was observed until the end of incubation (Fig. 4a). The RBC4 and SBC4 showed positive priming up to 2 days of incubation. The RBC6 followed by RBC4 showed higher negative priming of native SOC throughout the incubation period. The SBC4 and SBC6 showed almost similar negative priming during the incubation period. The MBC4 and MBC6 showed lower negative priming than the other BC, recording the lowest negative priming for MBC6. At the end of the experiment, RBC6 recorded the highest negative priming effect which was 20.4 times higher than the negative priming for MBC6.

In Inceptisol, all the BC except MBC6 exhibited positive priming effect on day 2, but after that MBC6 showed negative priming throughout the incubation period (Fig. 4b). RBC6 showed negative priming up to day 132, and thereafter it showed positive priming up to day 195 but again it showed negative priming for rest of the incubation period. RBC4 and SBC4 showed positive priming from day 2 to 25 and thereafter these showed negative priming for rest of the incubation period. MBC4 showed positive priming throughout the period. Among the BC, the negative priming was highest in SBC6, followed by RBC4, SBC4, MBC6 and RBC6. At 290th day of the incubation experiment, SBC6 recorded 5.48 times higher negative priming than RBC6.

In Mollisol, though RBC4 showed positive priming on day 2 and 7 but RBC6 showed positive priming until day 104; however, both the BC showed negative priming for rest of the incubation period (Fig 3c). Though SBC4 showed positive priming from day 2 to 74 and thereafter it showed negative priming for rest of the period; SBC6 showed negative priming throughout the incubation period ranging from day 2 to 290. Similarly, MBC4 showed positive priming from day 2 to 42 and thereafter negative priming for rest of the period, MBC6 showed negative priming throughout the incubation period. After 290 days of incubation experiment, it was revealed that the lowest negative priming was observed in MBC6 while RBC4 recorded the highest value (15.8 times higher than MBC6).

**Figure 5:**
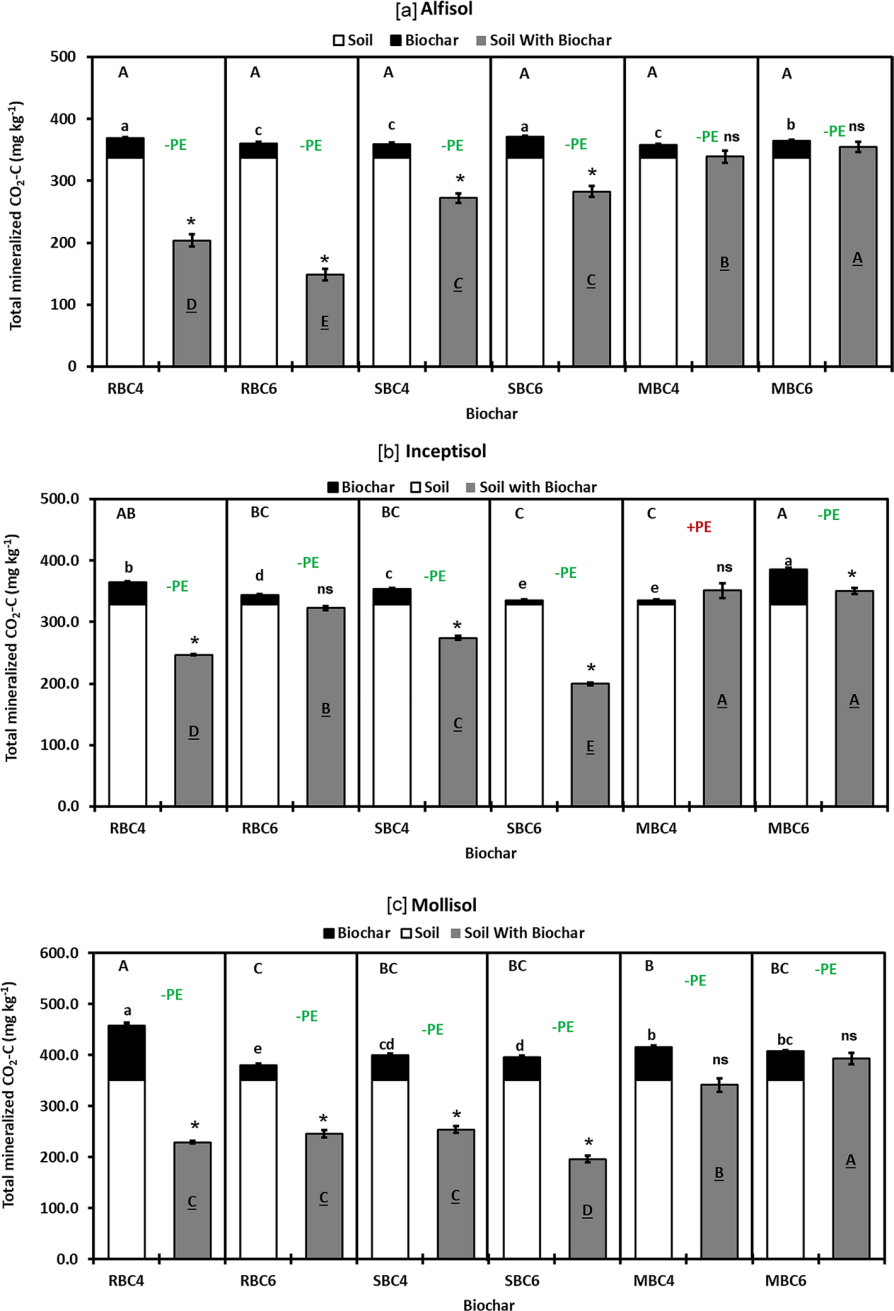
Total C mineralized in 290 days incubations of soil alone (S), biochar alone (Q-BC), and biochar mixed with (**a**) Alfisol, (**b**) Inceptisol and (**c**) Mollisol (S-BC). The histograms followed different upper case (sum of S and Q-BC i.e. S + Q-BC), lower case (Q-BC), and upper case underline (S with BC i.e. S-BC) are significantly different as per Duncan’s Multiple Range Test at *P* = 0.05; * indicates significant difference between sum of S and Q-BC (S + Q-BC) with S and BC mixture (S-BC) at P = 0.05 (Paired T test, n = 3 pairs) and ns indicates non-significant difference between these; Each histogram represents mean ± standard error (n = 3); RBC4-Rice husk biochar prepared at 400 °C; RBC6-Rice husk biochar prepared at 600 °C; SBC4-Sugarcane bagasse biochar prepared at 400 °C; SBC6-Sugarcane bagasse biochar prepared at 600 °C; MBC4-Mustard stalk biochar prepared at 400 °C; MBC6-Mustard stalk biochar prepared at 600 °C.

In priming experiment, the contribution to C mineralization from BC alone (Q-BC), soil alone (S), arithmetic sum of soil only and biochar only (S + Q-BC) and soil-biochar mixture (S-BC) was assessed to understand the effect of BC on native SOC priming. The extent and direction of BC induced priming on soil C varied across soil types. Among the BC only treatments (Q-BC) (Quartz with extracts from Alfisol), SBC6 being at par with RBC4 showed the highest C mineralization followed by MBC6 (Fig. 5a). Increase in pyrolysis temperature significantly decreased the C mineralization from RBC, while the trend was reversed for SBC and MBC. However, the S + BC treatments did not vary significantly. The C mineralization from S-BC mixture varied significantly across different BC treatments which followed the trend: S-MBC6 > S-MBC4 > S-SBC6 = S-SBC4 > S-RBC4 > S-RBC6. Increase in pyrolysis temperature significantly decreased the C mineralization from S-RBC mixture, while in the case of S-MBC mixture, it increased.

Among BC only treatments (Q-BC) (Quartz with extracts from Inceptisol), MBC6 showed the highest C mineralization followed by RBC4, and SBC4 (Fig. 5b). Increase in pyrolysis temperature significantly decreased the C mineralization from RBC and SBC, while for the MBC the reverse trend was noticed. The S + BC across all the BC treatments vary significantly and S + MBC6 being at par with S + RBC4 showed the highest C mineralization and S + SBC6 and S + MBC4 showed the lowest C mineralization. The C mineralization from S-BC mixture followed the trend: S-MBC6 = S-MBC4 > S-RBC6 > S-SBC6 > S-RBC4 > S-SBC6. The effect of pyrolysis temperature on C mineralization from S-BC mixture was BC specific, increase in pyrolysis temperature significantly increased C mineralization from S-RBC, while it decreased in the case of S-SBC.

Among BC only treatments (Q-BC) (Quartz with extracts from Mollisol), RBC4 showed the highest C mineralization followed by MBC4 jointly with MBC6 (Fig. 5c). Increase in pyrolysis temperature significantly decreased the C mineralization from RBC, while in the cases of SBC and MBC, the pyrolysis temperature did not have any influence on C mineralization. Among the S + BC treatments, RBC4 showed the highest C mineralization (Fig. 5c). The C mineralization from S-BC mixture followed the trend: S-MBC6 > S-MBC4 > S-SBC4 = S-RBC4 = S-RBC4 > S-SBC6. Increase in pyrolysis temperature significantly increased C mineralization from S-MBC mixture while in the case of S-SBC mixture it was reverse.

The paired T-test was carried out to study whether the C mineralization from S-BC mixture was different from the arithmetic sum of (S + Q-BC) or not. The pair mean data of the above two parameters were significant for RBC4, RBC6, SBC4, and SBC6 in both Alfisol (Fig. 5a) and Mollisol (Fig. 5c) and the MBC4 and MBC6 did not show significant differences in these soil orders. In Inceptisol, mixed results were obtained for RBC4, SBC4, SBC6, and MBC6 which showed significant differences and RBC6 and MBC4 did not show significant differences (Fig. 5b).

### Effect of biochar (BC) on pH, EC and available nutrients in soil

The application of MBC6, SBC6 and MBC4 significantly increased the pH of Alfisol (Table 3) to the tune of 11.4, 5.26 and 2.38% respectively. In Inceptisol and Mollisol, the highest pH was recorded with SBC6 though it was statistically at par with MBC6 in Inceptisol. The increase in pyrolysis temperature of MBC and SBC significantly increased of Alfisol and Mollisol pH while it was true for SBC in Inceptisol. The pH increments due to BC application varied from 0.04 to 0.67, 0.03–0.28, and 0.05–0.34 units in Alfisol, Inceptisol, and Mollisol, respectively. Overall, Alfisol showed the highest pH increment followed by Mollisol and Inceptisol. The EC increased due to BC application in all the soils, however it was at par with control in some of the BC treatments (Table 3). The EC was highest in MBC6 in all the three soils though it was statistically at par with MBC4 in Alfisol and MBC4, SBC6, RBC6 and RBC4 in Inceptisol. The increment in EC due to BC addition ranged from 0.01 to 0.18 units in Alfisol, 0.06–0.17 units in Inceptisol, and 0.02–0.13 units in Mollisol. The increase in pyrolysis temperature of SBC and MBC significantly increased the EC of Inceptisol (SBC6 increased EC of soil to the tune of 11.7% over SBC4) and Mollisol (EC enhanced by 23.3% for MBC6 over MBC4) respectively. Overall, the Alfisol showed the highest EC increment followed by Inceptisol and Mollisol.

**Table 3:** Effect of different biochar on pH and EC in three selected soils from India.

Treatment	Alfisol		Inceptisol		Mollisol	
	pH	EC (dS m^−1^)	pH	EC (dS m^−1^)	pH	EC (dS m^−1^)
RBC4	5.97^§cd^	0.30^c^	8.08^c^	0.83^ab^	7.65^b^	0.43^b^
RBC6	6.00^cd^	0.32^bc^	8.08^c^	0.86^a^	7.70^b^	0.42^b^
SBC4	5.93^cd^	0.30^c^	8.03^c^	0.77^b^	7.62^b^	0.43^b^
SBC6	6.20^b^	0.34^bc^	8.28^a^	0.86^a^	7.84^a^	0.43^b^
MBC4	6.03^c^	0.39^ab^	8.13^bc^	0.87^a^	7.55^c^	0.43^b^
MBC6	6.56^a^	0.47^a^	8.18^ab^	0.88^a^	7.70^b^	0.53^a^
Control	5.89^d^	0.29^c^	8.00^c^	0.71^c^	7.50^c^	0.40^b^

^§^The data followed by different lower case letters in a particular soil parameter in each column are significantly different according to Duncan’s Multiple Range Test at *P* = 0.05.

RBC4-Rice husk biochar prepared at 400 °C; RBC6-Rice husk biochar prepared at 600 °C; SBC4-Sugarcane bagasse biochar prepared at 400 °C; SBC6-Sugarcane bagasse biochar prepared at 600 °C; MBC4-Mustard stalk biochar prepared at 400 °C; MBC6-Mustard stock biochar prepared at 600 °C; EC-Electrical conductivity.

The available N content in Alfisol was recorded highest with SBC4 (98.5% higher than control) followed SBC6, MBC4 and RBC4/RBC6 (RBC4 and RBC6 are statistically similar) and MBC6 along with control showed the lowest value for available N content (Table 4). In Inceptisol, the SBC6 being at par with SBC4 and RBC4 showed the highest available N content which was 42.7% higher than control. In Mollisol, the RBC4 showed the highest available N content (80.8% higher than control) followed by SBC6/MBC4 (SBC6 and MBC4 are statistically similar), RBC6/SBC4 (RBC6 and SBC4 are statistically similar), MBC6 and control. Overall, the increase in pyrolysis temperature of BC significantly decreased the available N content in Alfisol for SBC and MBC, in Inceptisol for RBC, and Mollisol for RBC and MBC.

**Table 4:** Effect of different biochar on available nitrogen (AN), phosphorus (AP), and potassium (AK) contents three selected soils from India.

Treatment	Available N			Available P			Available K		
	Alfisol	Inceptisol	Mollisol	Alfisol	Inceptisol	Mollisol	Alfisol	Inceptisol	Mollisol
	(mg kg^−1^)								
RBC4	104§^d^	135^a^	158^a^	6.43^a^	11.9^a^	8.14^a^	93.1^b^	111^c^	41.9^d^
RBC6	103^d^	101^c^	126^c^	5.80^bc^	12.1^a^	8.41^a^	74.6^c^	118^c^	43.1^cd^
SBC4	162^a^	131^a^	123^c^	5.53^d^	11.8^a^	7.28^bc^	91.3^b^	147^b^	53.4^b^
SBC6	141^b^	137^a^	143^b^	5.23^d^	12.6^a^	7.91^ab^	95.7^b^	151^b^	54.5^b^
MBC4	129^c^	102^c^	139^b^	6.10^b^	13.6^a^	7.93^ab^	74.1^c^	111^c^	48.9^bc^
MBC6	84.4^e^	120^b^	111^d^	5.33^d^	12.2^a^	7.70^abc^	124^a^	193^a^	74.4^a^
Control	81.6^e^	96.0^c^	87.4^e^	4.63^e^	7.20^b^	7.03^c^	50.6^d^	73.8^d^	27.2^e^

^§^The data followed by different lower case letters in a particular soil parameter in each column are significantly different according to Duncan’s Multiple Range Test at *P* = 0.05.

RBC4-Rice husk biochar prepared at 400 °C; RBC6-Rice husk biochar prepared at 600 °C; SBC4-Sugarcane bagasse biochar prepared at 400 °C; SBC6-Sugarcane bagasse biochar prepared at 600 °C; MBC4-Mustard stalk biochar prepared at 400 °C; MBC6-Mustard stalk biochar prepared at 600 °C.

In Alfisol, RBC4 recorded the highest available P content followed by MBC4, RBC6 (Table 4) and recorded 38.9% more available P as compared to control. In Inceptisol, the available P content increased significantly due to the application of BC, whereas all the BC were at par with each other. In Mollisol, the RBC6 being at par with RBC4, MBC4, and SBC6 showed higher values for available P content. The effect of pyrolysis temperature of BC on decreasing the available P content was only evidenced in Alfisol for RBC and MBC but in Inceptisol and Mollisol the effect was not noticed.

The available K content was the highest in MBC6 followed by SBC4 and SBC6 (Table 4) among all the soils.Increase in pyrolysis temperature of MBC significantly increased the available K content only (Table 4). MBC6 increased available K by 2.45 times, 2.62 times and 2.74 times as compared to control inAlfisol, Inceptisol and Mollisol respectively.

### Principal component analysis

**Figure 6:**
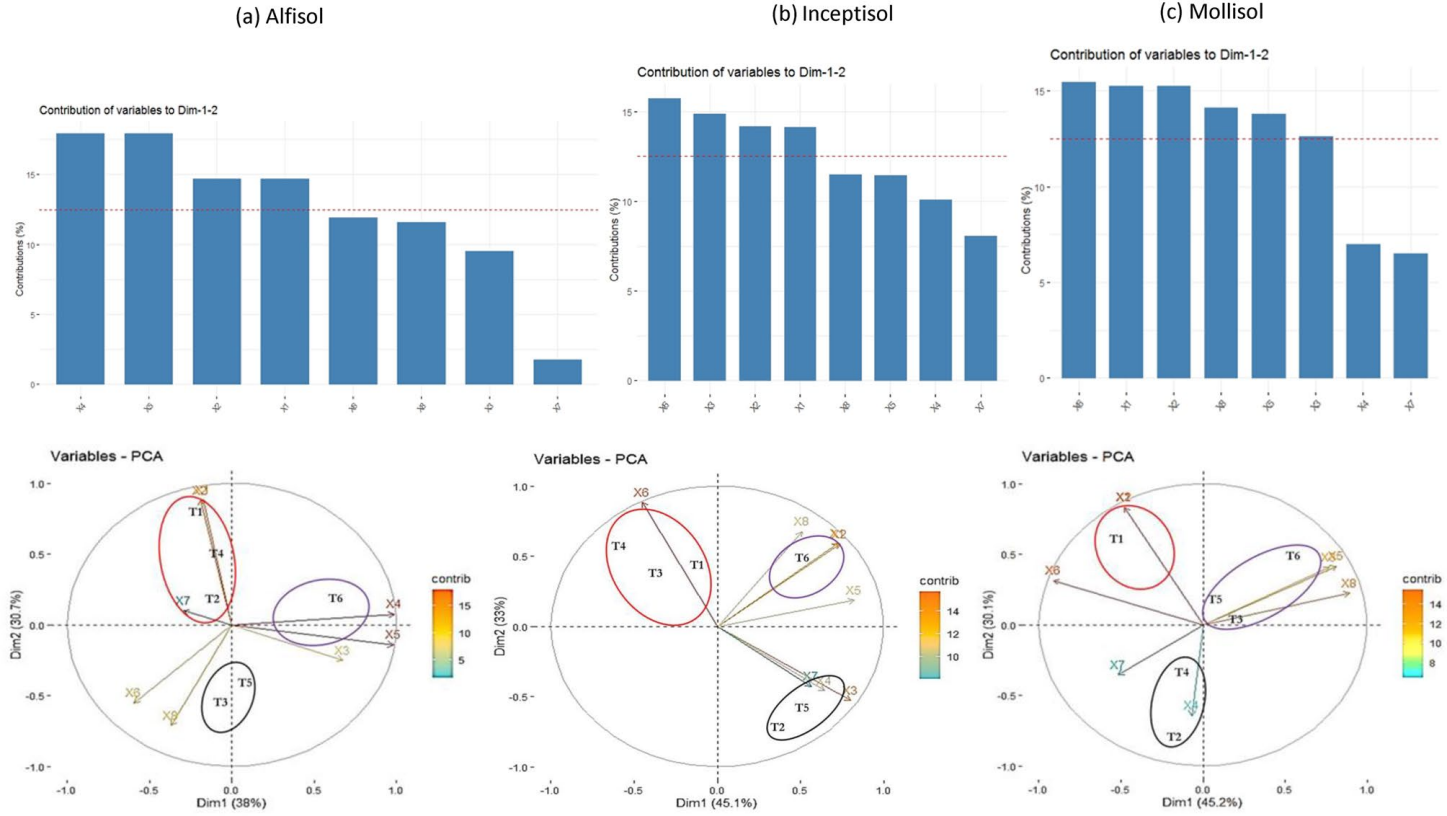
Identification of most important contributing parameters using PCA analysis, and PCA biplot cluster analysis for similarities of treatments and contributing parameters in (**a**) Alfisol, (**b**) Inceptisol, (**c**) Mollisol, X1-C mineralization biochar alone (Q-BC) X2-C mineralization soil + biochar (S + Q-BC), X3-C mineralization from soil-biochar mixture (S-BC), X4-pH, X5-EC, X6-available N, X7-available P, X8-available K, T1-RBC4, T2-RBC6, T3-SBC4, T4-SBC6, T5-MBC4, T6-MBC6. S-Control soil, BC-Biochar, Q-Quartz sand, RBC4-Rice husk biochar prepared at 400 °C; RBC6-Rice husk biochar prepared at 600 °C; SBC4-Sugarcane bagasse biochar prepared at 400 °C; SBC6-Sugarcane bagasse biochar prepared at 600 °C; MBC4-Mustard stalk biochar prepared at 400 °C; MBC6-Mustard stalk biochar prepared at 600 °C.

The principal component analysis (PCA) was performed to determine the contribution of different factors on priming of soil organic matter and identification of similar treatments in terms of BC feedstock and pyrolysis temperature (Fig. 6). The PCA biplot revealed first two principal components, together explaining 75.3% (45.2% in PC 1 and 30.1% in PC 2), 78.1% (45.1% in PC1 and 33% in PC2), and 68.7% (38% in PC1 and 30.7% in PC2) of the observed variability in Alfisol, Inceptisol, and Mollisol, respectively (Fig. 5a, b, and c). In Alfisol, the pH, EC, C mineralization from S-BC mixture and BC alone i.e. Q-BC emerged as the major variables (Fig. 6a). In the first cluster of biplot, MBC6 was the most significant treatment which had higher impact on soil pH, and EC and in the second cluster, RBC4, RBC6 and SBC6 had a significant impact on C mineralization from BC alone i.e. Q-BC and S + Q-BC. The third cluster with MBC4 and SBC4 did not have any impact on the studied parameters.

In Inceptisol, the available N, C mineralization from S-BC mixture and S + Q-BC, and BC alone i.e. Q-BC emerged as the most important parameters which were affected by different BC treatments (Fig. 5b). The biplot had three clusters, the first cluster identified as MBC6 which had measurable impact on C mineralization from BC alone i.e. Q-BC and S + Q-BC. The second cluster identified RBC4, SBC4, SBC6 had significant impact on available N content in soil. The third cluster, MBC4 and RBC6 had significant impact on C mineralization from S-BC mixture but might have negative impact on it.

In Mollisol, the available N and C mineralization from BC alone (Q-BC) and S + Q-BC, available K, EC, and C mineralization from S-BC mixture which were affected by BC treatments (Fig. 6c). The biplot had three clusters. In the first cluster, SBC4, MBC4, MBC6 had significant impact on C mineralization from S-BC mixture, EC, and available K content in soil. In the second cluster, RBC4 emerged as a treatment which significantly impacted C mineralization from S + Q-BC and BC alone (Q-BC).

## Discussion

### Biochar (BC) characteristics

The yield of BC was in consistent with that reported by others^53,68–70^. BC is enriched with C, and contains a range of plant macro, micro and secondary nutrient elements^53,71,72^. However, myriad of studies indicated that the composition, yield and other physico-chemical properties of BC depends upon the nature of feedstock and pyrolysis conditions^72–75^. Elevating the pyrolysis temperature has the potential to raise both pH and EC due to the concurrent increase in ash content, which includes inorganic components^76^ Thus, slow pyrolysis can, therefore, be characterized as a series of volatilization reactions that progressively leave behind an increasingly condensed carbonaceous matrix and ash with the loss of easily thermally degradable carbonaceous matter^77,78^. These observations align with our findings that increasing pyrolysis temperature enhanced pH and EC of most of the BC. The pH of SBC in the present study was alkaline in nature which increased the pH from 8.98 at 400 °C to 11.4 at 600 °C (Table 2). However, it was reported that the pH of SBC was acidic at 400°C which increased to 9.8 at 800 °C^79^. Moreover, SBC prepared at 400 and 600 °C recorded pH of 8.3 and 9.6, respectively^80^. This might be due to difference in production condition and biochemical composition of sugarcane variety^81^. Moreover, CO_3_^2^ and HCO_3_^−^ were the major alkaline components among the inorganic fractions responsible for the alkaline pH of BC^53^. The CCE of BC enhanced due to increase in ash content as evidenced from our experiment and other prior studies^53^. In addition, total C could be increased with increasing pyrolysis temperature since other elements, such as N are largely volatilized as temperature increases corroborating our findings^2^. Furthermore, increasing the pyrolysis temperature has the potential to enhance solid density, as the quantity of volatiles with low molecular weight decreases with rising temperatures^53,82^. At pyrolysis temperature of 600 °C, polyaromatic graphene sheets with high C concentration begins to grow at the expense of the amorphous C matrix, which forms below 300 °C^83^. Prior study reported that increase in pyrolysis temperature from 400 to 600 °C significantly increased the C content, while it decreased the N content in BC prepared from wheat straw, corn stover and switch grass except that was produced from rice hull^23^. These findings were in agreement with the other studies which also found higher C contents in plant material based BC, e.g., canola, soybean^84,85^, peanut shell, peanut hull, pine chips^29,86^, *Eucalyptus saligna* wood and leaf^87^. Overall, the SBC in our study was richer in C than either RBC or MBC (Table 2). Nevertheless, in line with our findings, it was reported that the C and N contents in rice hull BC prepared at 400°C as 55%, and 0.93%, respectively which were closer to our values^23^. The C content of SBC in our study was lower than that reported by others^79^. Nevertheless, it was suggested that rapid loss of O and H occurred between 300 and 500 °C^69^, our study aligns with this, revealing a swift decline in these elements in the BC between pyrolysis temperatures of 400°C and 600°C. This was the reason why H:C and O:C atom ratio of all the BC progressively decreased due to increase in pyrolysis temperature from 400 to 600 °C (Table 2) indicating more aromaticity in the BC prepared at higher pyrolysis temperature. The values of H:C and O:C atom ratio and the extent of decrease of the ratios are in conformity with the data published for wood and grass BC, pineapple leaves and sugarcane bagasse BC^69,80^. The observed increase in porosity with pyrolysis temperature in our study could be attributed to the gradual removal of volatiles from pores and the subsequent physical and chemical condensation of the remaining skeletal structure^88–90^. The increase in total and water-soluble P and K contents with increased pyrolysis temperature may be due to the higher concentration of these elements in the BC matrix. The difference in the content of P and K among the residues showed the effect of residue on BC nutrient properties similar to other studies^53,91^. Thus, depending on the requirement, residue and pyrolysis temperature could be chosen for tailoring BC suitable for specific applications in the soil to alleviate nutrient deficiency and improve soil productivity^92,93^.

### Biochar (BC) decomposition in soil

It has been proved from many studies that the addition of BC in soil caused an immediate flush of CO_2_ release^94,95^. After the short-term flush of C mineralization on application of BC to soil, there is evidence that BC is stable over periods of up to thousands of years^9,11^. The degree of aromaticity of BC also influences the BC stability in soil. The H, O, H:C atom ratio in BC emanated from our study decreased with increasing pyrolysis temperature (Table 2) which could be due to increased aromaticity in BC^53,96,97^. Moreover, the effect of pyrolysis temperature on total C mineralization, the three BC (RBC, SBC, MBC) displayed typical structural modifications of plant materials pyrolyzed at different temperatures. BC prepared at 600 °C had a more pronounced degree of aromaticity and a lower carboxyl group content, whereas BC prepared at 400 °C had a stronger residual contribution from structural polysaccharides for all three feedstocks as reported by others^39,98^. Furthermore, at the end of our incubation experiment, in soil-biochar mixture (S-BC), MBC6 showed higher decomposition. The aromaticity of MBC (mainly MBC6) of our study was comparatively lower than RBC and SBC as evidenced from the H:C and O:C ratio (Table 2) and this property might have played a significant role in supplying easily degradable C to continue fuelling accelerated microbial activities even at the later stages of decomposition. Besides, MBC being richer in N content, narrower in C:N and low ash content (Table 2) might have augmented increased rate of C mineralization throughout the incubation period. Nevertheless, a more aromatic structure and a higher C:N ratio (Table 2) would result in greater chemical recalcitrance and stability of RBC6 compared to RBC4 in Alfisol (Fig. 5a) and SBC6 compared to SBC4 in Inceptisol (Fig. 5b) and Mollisol in S-BC mixture (Fig. 5c)^53,99^. Here C mineralization from S-BC mixture did not exactly follow the same trend across three different soil types. Thus, soil type dictates the C mineralization pattern. Higher ash content in RBC6 (Table 2) probably made this BC more stable in Alfisol, while SBC6 with high aromaticity (Table 2) was the determining factor for its higher stability in both Inceptisol and Mollisol. In this context, it was reported that the same BC with special reference to wheat straw BC at 600 °C showed higher C mineralization when applied in an Ultisol with lower organic matter but showed lesser C mineralization in a Mollisol with higher organic matter^23^. The BC mineralization rates of our study are within the range of those reported by others in long-term incubation experiments in various soil types^17,22^. However, others measured much higher mineralization rates in Alfisol and Mollisol from different locations of the world^9,12,100,101^. The difference in BC decomposition rates may be mainly explained by the differences in BC properties due to the different pyrolysis temperatures.

### Biochar (BC) priming effect

In our study, the BC showed variable priming responses throughout the 290 days incubation period, and this was influenced by feedstock type, pyrolysis temperature, and more importantly soil type (Fig. 4a, b and c). The same BC behaved differently in different soils. Some researchers have suggested that the additional soil CO_2_ emission is an “apparent positive priming” effect within the first few days after the application of organic matter, which is linked to the accelerated turnover of native microbial biomass C rather than from the accelerated mineralization of native SOC^24^. BC generally results in short-term positive priming of native SOC, and longer-term C stabilization and these effects can be altered by global warming^13^. However, in our study, the positive C mineralization especially for MBC6 in Alfisol, MBC4 in Inceptisol, and RBC6 in Mollisol extended for 100 days or more periods which suggested priming of native SOC to C mineralization (Fig. 4a, b and c). As the pH of MBC6 was highly alkaline (Table 2), its application increased the pH of the Alfisol from 5.90 (Table 1) to 6.56 (Table 3) and thereby augmented the microbial activities leading to positive priming of native SOC in the first phase. Moreover, the liming effect of MBC6 was highest as evidenced from the calcium carbon equivalent value (Table 2) which corroborated our findings. Additionally, priming effect of MBC6 estimated over 290 days was significant as evidenced from PCA analysis (Figs. 5a and 6a). On the contrary, MBC4 having pH of 8.47 (Table 2) increased the pH of Inceptisol from 8.00 (Table 1) to 8.13 (Table 3) and having moderate volatile matter content (Table 2) probably contributed to positive priming of SOC though it was not significant as compared to the arithmetic sum of C mineralization from soil and MBC4 (S + MBC4) (Fig. 5b) In Mollisol, MBC6 behaved similarly to that in Alfisol. Nevertheless, in our experiment same BC exhibited diverse priming response in different soils corroborating soil properties play important role in priming of native SOC by BC. For instance, in Inceptisol RBC6 showed positive priming for longer period (195 day) as compared to Mollisol (104 day) (Fig. 4b and c) might be due to higher SOC content in Mollisol (8.58 g kg^−1^) (Table 1). However, in long run, RBC4 as well as RBC6 showed negative priming effect on native SOC. Similar type of observation over 60 days period was reported with respect to corn stover BC at 600 °C having volatile matter content 14.1% applied to Mollisol with higher SOC content (26.8 g kg^−1^)^23^. Moreover, prior literature has shown that *Eucalyptus saligna* wood BC prepared at 500 °C caused smaller positive priming in the clay-poor Inceptisol or negative priming in the clay-rich Entisol, Oxisol and Vertisol^30^. In line with this, we also noticed similar type of observation in clay rich Mollisol with higher OC content (8.58 g kg^−1^) (Table 1) which showed shorter positive priming effect than the clay poor and low OC containing Alfisol (5.14 g kg^−1^) (Table 1) and Inceptisol (4.11 g kg^−1^) (Table 1).

However, irrespective of soil orders, some BC showed significantly higher negative priming effect from S-BC mixture (Fig. 5a, b and c). In this context, given the reduced liming effect of RBC6 (with low CCE, Table 2) compared to other BC, the increase in pH in the acidic Alfisol was lesser (Table 3). This could have contributed to a suppression of microbial activities, potentially leading to an intensified negative priming effect of native SOC (Fig. 5a). At the end of incubation experiment, the highest negative priming in S-BC mixture for RBC6 and lowest CumC_min_ from S-RBC6 also supported our finding (Figs. 3a and 4a). Nevertheless, as the aromaticity of SBC6 was the lowest as evidenced from the lowest O:C ratio (Table 2), it showed negative priming by depressing the decomposition of native SOC in Inceptisol and Mollisol (Fig. 5b and c). Besides, probably higher C:N ratio of SBC6 (C:N::81:1) (Table 2) might have depressed the decomposition of soil organic matter further. Therefore, the difference in CumC_min_ between S + SBC6 and S-SBC6 mixture was higher than the same combination with respect to SBC4 in Inceptisol and Mollisol (Fig. 5b and c). Similar trend was also found in CumC_min_ from S-BC mixture in case of SBC6 Inceptisol and Mollisol (Fig. 3b and c) corroborating our findings. All the soil orders of our study showed negative priming with varying magnitudes which were BC specific. Similar to the present study, a positive and negative priming effect was also reported by others^93,102–104^. The pyrolysis temperature did not show any specific trend with respect to quantity and the direction of priming across feedstock and soil type. Though prior study reported that Eastern gamma grass (*Tripsacum dactyloides* L.) BC, produced at lower temperature (250 and 400 °C), demonstrated a positive priming effect during the early incubation stages (90 days) when applied to soils with low SOC content. However, a contrasting negative priming effect emerged during the later incubation stages (250–500 days) when high-temperature hardwood BC (produced at 525 and 650 °C) was applied to soils with high SOC^12^. Probably the BC characteristics e.g., alkalinity, volatile matter content, CCE, aromaticity, C:N might have interacted with the soil organic matter for deciding the magnitude and direction of priming effect.

### EC, pH, and available nutrients in soil

The liming properties of BC are achieved by the presence of alkalinity in BC and its higher calcium carbonate equivalent. MBC6 had the highest liming properties as evidenced by enhanced pH and EC in all the soil orders (Table 3). High alkalinity, CCE and EC of MBC6 (Table 2) were the main driving force for enhancing the pH of Alfisol and Inceptsol (Table 3). Overall, the high temperature BC had higher pH (Table 2) and therefore their applications in soil enhanced the pH of the soil (Table 3). Along with pH, the associated changes in soil electrical conductivity (EC) were also noticed especially in the case of MBC which itself showed the highest EC (Table 2). The effect of pyrolysis temperature on a significant increase in pH was only noticed in MBC, SBC in Alfisol and Mollisol, and SBC in Inceptisol (Table 3). After reviewing and compiling the world literature, it was concluded that the BC could increase the soil pH by 0.1–2.0 units in a wide range of soils varying in native pH^72^. It was indicated that the magnitude of soil pH change upon BC addition was inevitably reliant on soil types, BC properties, and application rates. The green waste BC and poultry litter BC could gradually increase pH by 0.6–2.0 units of an acidic Alfisol at successive application rates ranging from 10 to 100 Mg ha^−1^ under radish (*Raphanus sativus*)^44,45^. Moreover, it was observed that BC (pH 9.4) at the rate of 50 and 100 Mg ha^−1^ enhanced pH of Alfisol and as a result, exchangeable Al concentration was also reduced in soil^105^. Contrarily, it was reported that the maximum pH increment was noticed in Alfisol with the BC application rate of 25–50 Mg ha^−1^. As the BC application rate of our study was 10 Mg ha^−1^, therefore at this rate of BC application was effective only for neutralization of active and potential acidity and therefore the percent pH change was low as compared to two other soils having slightly alkaline in reaction. Moreover, PCA biplot analysis revealed that MBC6 was the most significant treatment having higher impact on soil pH, and EC in Alfisol (Fig. 6a) which corroborated our findings of incubation experiment.

Overall, the higher available N in SBC4 and RBC4 treated soil was due to stimulation of gross N mineralization, recalcitrant N fraction and labile N fraction^72,106,107^. Besides, the associated increase in pH of acid soil due to BC addition also helped in increased availability of primary and secondary nutrients like N, P, and K^46,48,108–111^. The application of BC significantly influences the mineralization-immobilization turnover of nutrients, which is affected by altering both microbial activities and the community structure of soils. The higher C:N ratio of BC when applied to soil, might trigger to decompose the native soil organic matter (SOM) to acquire N via priming effect^24,112^. In our study except MBC4, other BC showed negative priming of SOC across all the soil orders assessed over 290 days but immobilization of neither N nor P was noticed. Thus, most of the mineralized NH_4_^+^ under BC treatments probably came from the recalcitrant N in soil, while in the control soil most mineralized NH_4_ originated from the labile N^101^. Another reason could be due to the BC induced modifications of soil porosity/aeration that stimulates the aerobic/heterotrophic microbial population resulting in the degradation of recalcitrant SOM especially during the first 100 days of incubation in the presence of BC. The high H:C ratio in RBC4 and SBC4 (Table 2) also might have triggered the gross N mineralization^113^. Moreover, PCA analysis also identified RBC4, SBC4, SBC6 had significant impact on available N content in Inceptisol (Fig. 4b) which further supported the impact of RBC4, SBC4 on higher N availability in soil.

Most of the BC showed negative priming of native SOC in long run (290 days) though it was not necessarily reflected in priming of available N, P contents in soil. This could be due to mineralization-immobilization turnover of N and P in soil which might have caused positive net mineralization of these nutrients. Besides, BC being a source of N and P, might have contributed to inorganic pool even though it was reported to show negative priming of organic C in an Inceptisol^70,114^.

In spite of the negative priming of SOC, all these BC enhanced the available N, P, and K contents in soil. Aligning with our findings, prior study documented that the application of carbonized rice husks increased total N, available P and K in rice growing soil of Philippines^115^. Moreover, it was also reported rice husk BC was also reported to enrich soil fertility and improve rice growth^94^. There are also reports on significant increase in water-soluble P in acidic silt loam, clay loam, and an intermediate loam soil treated with 1% wheat BC^114^ and 20- and 28-folds increase in Mehlich 1 extractable P due to BC application^35^. The mechanism of increased P availability might be due to desorption of P from adsorbed P on ferrihydrite due to presence of BC^52^. Additionally, a comprehensive review of international literature and a subsequent meta-analysis revealed that BC significantly increased "available P by 45%" in the surface soil. The increased availability of K (Table 4) in all the soil orders due to application of MBC6 was mainly due to its higher water-soluble K contents (Table 2). On the other hand, K, being a non-structural element in plant mainly present in water-soluble and exchangeable form in soil which does not take part in mineralization-immobilization turn over. Therefore, the K content in BC largely influenced the available pool of K. MBC6 being rich in water-soluble K, significantly increased available K content in all the soil orders (Table 4) and this (except Alfisol) was clearly evidenced in PCA biplot analysis which also showed the association of this treatment positively impacted the available K content (Fig. 6a, b, and c).

## Conclusion

The physical and chemical properties of BC are greatly influenced by feedstock type and pyrolysis temperature. Decomposition of BC followed a different trend across different soil types. MBC6 in Alfisol and RBC6 in Mollisol fuelled the microbial activities up to 104 days, while MBC4 showed up to 290 days. The pyrolysis temperature did not show clear trend with respect to priming effect across feedstock and soil type. Therefore, the C retention potential of BC was found to be soil order specific. Thus, the RBC6 in Alfisol, SBC6 both in Inceptisol and Mollisol recorded the lowest C mineralization of native SOC which strongly suggest their potential for enhanced C retention. Most of the BC showed negative priming of native SOC in long run (290 days) but all these BC enhanced the available N, P, and K contents in soil. Therefore, application of BC is a win–win strategy to preserve native SOC and improves soil fertility. When BC is considered as an amendment for enhancing soil fertility, SBC4 could be advocated for increasing N availability in both Alfisol and Inceptisol, while RBC4 for enhancing N and P availability in Mollisol, and P availability in Alfisol. Furthermore, MBC6 emerged as a promising amendment for the reclamation of acidic Alfisol and enhancing K availability across diverse soil orders. It is evident that a single BC may not fulfil all our important management goals e.g., C sequestration, nutrient availability, and amelioration of acid soil. This study clearly indicated that depending upon the management goal, tailor made BC could be developed for its use as a soil amendment.

## Data Availability

The datasets used or analyzed during the current study are available from the corresponding author on reasonable request.
